# Smoking increases expression of the SARS-CoV-2 spike protein-binding long *ACE2* isoform in bronchial epithelium

**DOI:** 10.1186/s12931-023-02430-5

**Published:** 2023-05-11

**Authors:** Simon D. Pouwels, Maarten van den Berge, Gwenda F. Vasse, Wim Timens, Corry-Anke Brandsma, Hananeh Aliee, Pieter S. Hiemstra, Victor Guryev, Alen Faiz

**Affiliations:** 1 Department of Pathology & Medical Biology, University of Groningen, University Medical Center Groningen, Groningen, The Netherlands; 2 University of Groningen, University Medical Center Groningen, Groningen Research Institute for Asthma and COPD, Groningen, The Netherlands; 3 Department of Pulmonary Diseases, University of Groningen, University Medical Center Groningen, Groningen, The Netherlands; 4 European Research Institute for the Biology of Ageing, University of Groningen, University Medical Center Groningen, Groningen, The Netherlands; 5 Respiratory Bioinformatics and Molecular Biology (RBMB), School of Life Sciences, University of Technology Sydney, Building 4, Room 04.07.418, Thomas St, Ultimo, NSW 2007 Australia; 6 Institute of Computational Biology, Helmholtz Centre, Munich, Germany; 7 Department of Pulmonology, Leiden University Medical Center, Leiden, The Netherlands

**Keywords:** COVID-19, SARS-CoV-2, ACE2, Cigarette smoking

## Abstract

After more than two years the COVID-19 pandemic, that is caused by infection with the respiratory SARS-CoV-2 virus, is still ongoing. The risk to develop severe COVID-19 upon SARS-CoV-2 infection is increased in individuals with a high age, high body mass index, and who are smoking. The SARS-CoV-2 virus infects cells of the upper respiratory tract by entering these cells upon binding to the Angiotensin-converting enzyme 2 (ACE2) receptor. ACE2 is expressed in various cell types in the lung but the expression is especially high in goblet and ciliated cells. Recently, it was shown that next to its full-length isoform, *ACE2* also has a short isoform. The short isoform is unable to bind SARS-CoV-2 and does not facilitate viral entry. In the current study we investigated whether active cigarette smoking increases the expression of the long or the short ACE2 isoform. We showed that in active smokers the expression of the long, active isoform, but not the short isoform of *ACE2* is higher compared to never smokers. Additionally, it was shown that the expression of especially the long, active isoform of ACE2 was associated with secretory, club and goblet epithelial cells. This study increases our understanding of why current smokers are more susceptible to SARS-CoV-2 infection, in addition to the already established increased risk to develop severe COVID-19.

## To the editor:

The currently ongoing COVID-19 pandemic, caused by infection with the respiratory SARS-CoV-2 virus, has led to 469 million reported cases and 6 million deaths worldwide in less than 2 years (World Health Organization, 22 March 2022). The most important risk factors for the development of severe COVID-19 upon SARS-CoV-2 infection include high age, high body mass index, and active smoking. Although smokers have an increased risk of developing severe COVID-19, the effects of cigarette smoke are complex as some studies show that the infection risk by SARS-CoV2 is equal or even decreased in active smokers [1–3]. SARS-CoV-2 infects cells of the upper respiratory tract by entering these cells upon binding of the spike protein to the Angiotensin-converting enzyme 2 (ACE2) receptor. ACE2 is expressed in a number of cell types in the lung, but especially highly expressed in goblet and ciliated cells [4]. Of interest, it was recently shown that along with its full-length isoform, *ACE2* also has a short isoform. This newly discovered short isoform lacks the high affinity spike protein S1 binding site located on exon 2, preventing the binding to SARS-CoV-2 and is thus not involved in facilitating viral entry [5, 6]. This, together with recent observations that smoking is associated with higher expression of *ACE2* in lungs, [7] puts forward an important question – does active cigarette smoking increase the expression of the long or the short ACE2 isoform, or both?

To investigate whether active smoking is associated with higher expression of the short or long isoform of *ACE2*, we examined the expression of these *ACE2* isoforms in bronchial biopsies from never-smokers and current smokers. The Study to Obtain Normal Values of Inflammatory Variables From Healthy Subjects (NORM; NCT00848406) included asymptomatic smokers and never-smokers with normal lung function. All participants provided informed consent and the study was approved by the Medical Ethical committee of the University Medical Center Groningen (UMCG), The Netherlands. Study subject characteristics are shown in Table 1. Bronchial biopsies were collected and processed for RNA-Seqecuencing as previously described [8]. Bulk RNA-seq data has been deposited at the EGA (accession number: EGAS00001003735). scRNA-Seq signatures of 15 cell types from our previously-published data were utilized to determine differences in cell-type composition based on mRNA expression levels [9]. Due to highly similar gene expression profiles, the scRNA-Seq signatures from the club and the two goblet cell clusters were combined to generate a uniform scRNA-Seq signature of secretory cells [10].

**Table 1:** Study subject characteristics

	Current smokers	Never smokers
**n**	37	40
**Mean Age (SD)**	41.6 (15.2)	38.5 (18.9)
**Gender male n(%)**	22 (59.5)	20 (50)
**Mean Pack years (SD)**	18.8 (15.1)	0 (0)
**Mean FEV1% (SD) Prediction**	99.3 (9.2)	101.5 (11.9)
**Mean FEV1/FVC (SD)**	78.2 (6.1)	80.7 (6.8)

**Fig. 1:**
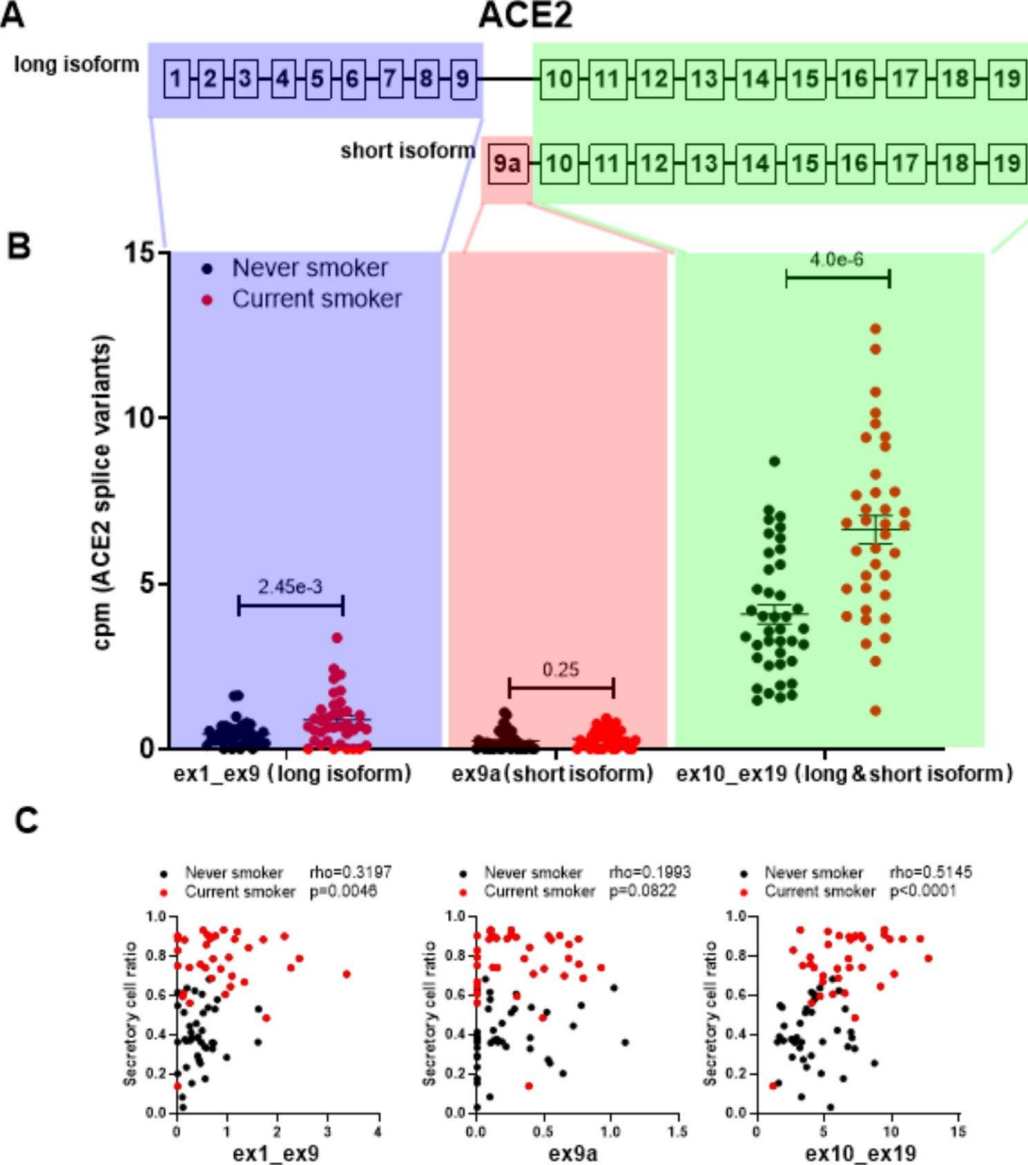
Influence of smoke exposure on *ACE2* expression. **(A)** Diagram of the *ACE2* isoforms as decribed previously [6]. **(B)** Influence of smoking status (current n = 37 vs. never smokers n = 40) on counts per million reads mapped (CPM)-normalised *ACE2* isoform expression in bronchial biopsies, mean ± SEM (Wilcoxon signed-rank test). Read counts corresponding to exons 1–9 and 9a-19 were calculated using the htseq-count tool of HTSeq package v.0.12.4. The number of reads mapping to exon 9a was calculated separately as reads overlapping genomic range of exon 9a – GRCh38:chrX:15,580,281 − 15,580,438. **(C)** Association of CPM-normalised *ACE2* isoform expression with the estimated proportion of secretory cells (n = 77; Spearman test)

By quantifying the reads from exons 1–9 (specific for the long isoform of *ACE2*), we can selectively investigate the long isoform (Fig. 1A). Here, a significant increase in the expression of the long isoform of *ACE2* was observed in current smokers compared to never smokers (Fig. 1B). In contrast, no differences were observed in the expression of the shorter isoform (exon 9a), however the expression of this isoform was rather low. For exons 10–19, shared between the two isoforms, a significantly higher expression was observed in smokers compared to never smokers, which may be attributed to the more abundant long isoform.

We and others have shown that *ACE2* expression is higher in club and goblet cells compared to other cells in bronchial biopsies [4]. Previously, we have shown that the increased expression of *ACE2* in active smokers is associated with goblet cell hyperplasia [7]. To confirm that both isoforms are higher expressed with goblet cell hyperplasia, we correlated the abundance of secretory (club and goblet) cells, obtained by deconvolution analysis, with the expression of these isoforms. Here, we found that the expression of the long isoform of ACE2 correlated with the percentage of secretory cells, while a trend was observed for the short isoform (Fig. 1C). Exons 10–19 that are shared between the two isoforms were strongly correlated with secretory cells.

The current study shows that smoking is associated with higher expression of the long, active isoform of *ACE2*, whereas this association does not exist for the short isoform. Of note, our study only assessed the gene expression levels of *ACE2*, which may not always directly reflect the protein levels. Higher expression of this SARS-CoV-2 spike protein-binding isoform of ACE2 may render current smokers more susceptible to SARS-CoV-2 infection, in addition to the already established increased risk to develop severe COVID-19. Future studies using overexpression models of the long ACE2 isoform should confirm the function of this isoform in SARS-CoV2 infections.

## Data Availability

The datasets used and/or analyzed during the current study are available from the corresponding author on reasonable request.
